# Supplementary material for: A Global Screen for Assembly State Changes of the Mitotic Proteome by SEC-SWATH-MS
Source: Cell Syst. 2020 Feb 26;10(2):133–155.e6. doi: 10.1016/j.cels.2020.01.001 (PMC7042714; doi:10.1016/j.cels.2020.01.001)

Q8IXM3 | RM41_HUMAN | MRPL41 BMRP MRPL27 RPML27 PIG3
Monomer MW [kDa]: 15.383 Monomer expected elution fraction: 57

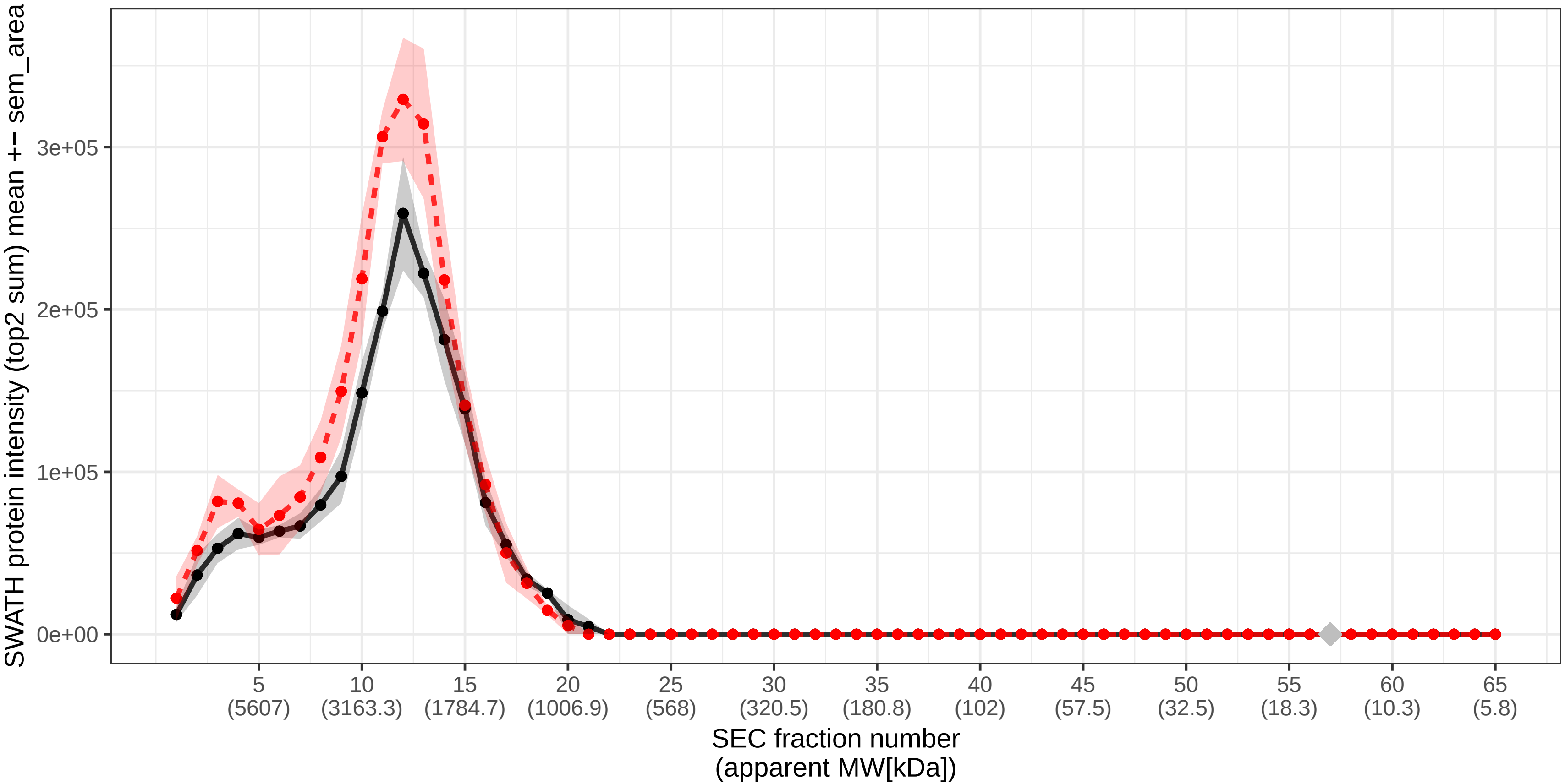

Supplement: Data S1. SEC-SWATH-MS Protein Chromatograms, Related to Figure 1 [file mmc6.zip › SECchrom_Q8IXM3_RM41_HUMAN_MRPL41_BMRP_MRPL27_RPML27_PIG3.pdf]
